# Stress and Cardiac Arrhythmias

**DOI:** 10.1016/s0972-6292(16)30793-8

**Published:** 2014-10-06

**Authors:** Beena Johnson, Johnson Francis

**Affiliations:** 1 Senior Consultant in Child Guidance and Psychosomatic Medicine, Baby Memorial Hospital, Calicut, Kerala, India; 2 Senior Consultant Interventional Cardiologist, Baby Memorial Hospital, Calicut, Kerala, India

**Keywords:** Stress, Cardiac Arrhythmias

Stress can exert adverse effect on cardiovascular health. Psychosocial stress adversely affects the autonomic homeostasis. This in turn can result in metabolic abnormalities, inflammation and dysfunction of endothelium [1]. Changes in the autonomic homeostasis can be a major trigger for ventricular tachyarrhythmias [2]. Increased sympathetic nervous activity can cause increased proarrhythmic repolarization instability leading to spontaneous ventricular arrhythmias. During stress-induced autonomic nervous system activity, the heart rate rises and the heart rate variability indices like *low frequency power* falls before the onset of ventricular tachycardia [3]. Psychological stress has been shown to induce T wave alternans, which in turn predicts future ventricular tachyarrhythmia events [4]. Fluctuations in T wave amplitude after psychological stress are predictive of subsequent arrhythmic events [5].

The mechanism of arrhythmia in children with structurally normal heart is the same as in an adult patient. Arrhythmias in children with heart disease can be the result of any underlying structural abnormality. It may also be due to surgical interventions [6]. Psychological stress stimulate sympathetic nervous system and this in turn can become proarrhythmic [7].

Catecholaminergic ventricular tachycardia is a rare primary ventricular tachyarrhythmia seen in children, which has a poor natural history. This potentially lethal tachyarrhythmia in children with structurally normal heart can be induced by stress or emotions [8].

Myocardial electrical instability can be triggered by psychological stress. Chronic stress can lead to reduced heart rate variability, increased QT dispersion and reduced baroreceptor sensitivity. Patients with greatest changes in the cardiac neural regulation associated with increased sympathetic activity due to stress have the greatest risk for developing fatal ventricular arrhythmias [9].

The influence of anxiety on cardiovascular disease is well known. Both chronic and acute psychosocial stressors leading to anxiety are associated with arrhythmic risk. Out of the 96 studies related to psychosocial aspects of arrhythmic risk conducted among different populations, 92% of the studies demonstrated positive correlation between psychosocial stressors and arrhythmias [10]. Emotional stressors can lead to ventricular ectopic beats and ventricular tachycardia. Though disturbances of cardiac rhythm due to emotional stress are often transient, sometimes the consequences can be seriously damaging and even fatal [11].

The importance of psychological factors in the genesis of cardiac arrhythmias is well recognized. Among the emotional factors, anger is strongly associated with triggering of ventricular arrhythmias [12]. Negative emotions like anger and hostility increases the risk of atrial fibrillation [13]. Depressive symptoms can predict arrhythmic death [14]. Depression and anxiety act through common pathway leading to increased arrhythmic risk [15].

Sudden emotional arousal can even trigger malignant ventricular arrhythmias. It is estimated that about 20 - 40% of sudden cardiac deaths are precipitated by acute emotional stressors [16]. Cardiac autonomic dysfunction triggered by psychological distress can increase the risk of arrhythmias [12]. With the advent of functional neuro imaging, the anatomical substrate and the physiological mechanism by which emotional stress contributes to the arrhythmias and cardiovascular events are now recognized. During emotional stress there is lateralization of cerebral activity. This leads to asymmetrical stimulation of the heart, producing areas of inhomogeneous repolarization, creating electrical instability. This in turn facilitates the development of cardiac arrhythmias.

Non pharmacological approaches like relaxation therapy will be beneficial in the management of emotional stress [11]. As stress and negative emotions are important risk factors in arrhythmogenesis, to reduce the risk of arrhythmia in patients with psychological distress, stress management has a very important role.
